# Editorial: Herpesviruses of animals: recent advances and updates

**DOI:** 10.3389/fvets.2023.1326282

**Published:** 2023-11-07

**Authors:** Selvaraj Pavulraj, Walid Azab

**Affiliations:** ^1^Department of Pathobiological Sciences, School of Veterinary Medicine, Louisiana State University, Baton Rouge, LA, United States; ^2^Institut für Virologie, Robert von Ostertag-Haus, Zentrum für Infektionsmedizin, Freie Universität Berlin, Berlin, Germany

**Keywords:** feline herpesvirus, equine herpesvirus, autophagy, Marek's disease, mobile genetic element, common buzzard

The Herpesviridae are a large family of enveloped, double-stranded DNA viruses found in many different orders of vertebrates, including birds, reptiles, amphibians, fish, and mammals (1). These viruses have a long evolutionary history of co-evolution with the host (2). In the end, the viruses have reached a fine-tuned balance with their corresponding host, which allows them to persist and spread successfully to new hosts, either of the same species without being lethal or to new hosts where they are most likely lethal (3, 4). The diseases associated with herpesviruses range from mild skin lesions, respiratory and reproductive problems, and neurological disorders to tumors and death. Primary herpesvirus infection usually results in productive infection, which is subsequently limited by host immune responses, leaving behind a lifelong latent infection. Latency is the state in which virus genome is carried within the cells in the absence of virus production but with the ability to reactivate and re-enter the lytic cycle (5). The delicate balance between herpesviruses and hosts results from the interaction of a variety of viral and cellular factors, which together shape the tropism for the host. Different strategies of virus pathogenesis have been documented (6–8); several are yet to be discovered. Understanding these interactions will provide insights into the viral life cycle and cell biology in general. Furthermore, this research will facilitate comprehension of herpesvirus pathogenesis and enable the development of new strategies to combat disease-causing herpesviruses. The successful development of therapeutic vaccines and antiviral drugs are dependent on understanding the interactions between virus and host cell factors. This Research Topic focuses on new advancements on virus-host interaction, virus pathogenesis, emerging virus infection, and novel therapeutics.

Feline herpesvirus type 1 (FeHV-1) is an important viral disease of cats causing feline viral rhinotracheitis. The virus belongs to the genus Varicellovirus, subfamily Alphaherpesvirinae in the Herpesviridae family (9). Autophagy is a conserved cellular process leading to sequestration and degradation of intracellular components and foreign material including viruses in cytosolic organelles. Herpesviruses use several strategies to evade, resist, or exploit the autophagy network to their advantage, and promote viral proliferation (10). However, the role of FeHV-1 and its relationship with the autophagic process has not yet been elucidated. Ferrara, Sgadari et al. evaluated the autophagy mediated by FeHV-1 and determined its proviral role. Autophagy is induced by FeHV-1 in a viral dose-dependent manner, *in vitro*. The pro-viral role of autophagy during FeHV-1 infection was established using late autophagy inhibitors and inducers, by assessing the viral yield, cytotoxic effects, and expression of viral glycoproteins. Autophagy inhibitors negatively impact viral replication. Furthermore, the autophagy inducer showed increased accumulation of glycoprotein B, a viral protein in the treated cells. In summary, this study demonstrates FeHV-1-mediated autophagy induction, its proviral role, and the negative impact of late autophagy inhibitors on viral replication.

The PI3K/Akt/mTOR axis is a key regulator of many cellular processes including translation, metabolism, autophagy, and cell death, and can fulfill many viral requirements (11). In the follow-up study, Ferrara, Longobardi et al. elucidated the involvement of this pathway during FeHV-1 infection in permissive cell lines. The expression of the proteins involved in the PI3K/Akt/mTOR pathway was determined by immunoblotting in the FeHV-1-infected cells. FeHV-1 infection modulated the expression of several proteins of the PI3K/Akt/mTOR pathway, suggesting that FeHV-1 may interact independently with different autophagic signaling pathways. Early phosphorylation of Akt also indicates a functional role for this axis in viral entry. However, Akt knockdown did not show any reduction in viral replication. The presence of a protein kinase in the FeHV-1 genome (encoded by the Us3 gene) might have phosphorylated various Akt substrates as an Akt surrogate, as reported in herpes simplex virus type 1 and pseudorabies virus infection (HSV-1 and PRV). The study highlights changes in the PI3K/Akt/mTOR pathway during FeHV-1 infection.

Equine herpesvirus type 1 (EHV-1) is a major viral pathogen of equines causing respiratory disease, abortion at the last trimester, neonatal foal mortality, and chorioretinopathy. The EHV-1 belongs to the genus Varicellovirus in the subfamily Alphaherpesvirinae (12). A major sequela that can occur after an EHV-1 infection is a neurological disease termed equine herpesvirus myeloencephalopathy (EHM), characterized by ataxia, incontinence, and partial to full paralysis, which may ultimately lead to the euthanasia of the infected horse (13). Understanding virus-host interaction in the EHM pathogenesis is critical to developing an effective therapeutic strategy for EHM. Black and Frampton studied the ability of four drugs (i) non-steroidal anti-inflammatory drugs (NSAIDs; flunixin meglumine), (ii) a steroidal anti-inflammatory drug (dexamethasone), (iii) a Rho-kinase (ROCK) inhibitor, and (iv) a JAK/STAT inhibitor (AG490) to reduce EHV-1 virus yields and cell-to-cell spread, *in vitro*. Among the four drugs, Flunixin meglumine and AG490 (JAK/STAT inhibitor), significantly reduced virus yields, and cell-to-cell virus spread in endothelial and epithelial cell lines.

Mobile genetic elements (transposable elements and plasmids) and viruses display significant diversity with various life cycles, but how this diversity emerges remains obscure (14). A novel giant mobile element, Teratorn, was identified in the genome of medaka, Oryzias latipes. Teratorn is a composite DNA transposon created by a fusion of a piggyBac-like DNA transposon and a novel herpesvirus of the Alloherpesviridae family (15). A genomic survey revealed that Teratorn-like herpesviruses are widely distributed among teleost genomes, the majority of which are also fused with piggyBac, suggesting that fusion with piggyBac is a trigger for the life-cycle shift of authentic herpesviruses to an intragenomic parasite. Thus, Teratorn-like herpesvirus provides a clear example of how novel mobile elements emerge. The review by Inoue and Takeda, discussed the unique sequence and life-cycle characteristics of Teratorn, followed by the evolutionary process of piggyBac-herpesvirus fusion based on the distribution of Teratorn-like herpesviruses among teleosts. Finally, they proposed that recombination could be a driving force generating novel mobile elements.

Marek's disease (MD) is a highly contagious viral disease of chickens characterized by T-cell lymphomas and peripheral nerve enlargement caused by the MD virus (MDV) and belongs to the genus Mardivirus in the subfamily Alphaherpesvirinae (16). The virus establishes latency in chicken T lymphocytes that can lead to T cell transformation and tumor. Transformed MD chicken cell lines (MDCCs) are a valuable *ex vivo* model to study MDV latency, transformation, and reactivation (17). Tien et al. developed MDCCs from chickens infected with MDV that fluoresce during lytic replication and reactivation. Treating MDCCs with Sodium butyrate increased MDV reactivation; however, it caused significant apoptosis and necrosis. In contrast, treatment of MDCCs by decreasing the temperature resulted in robust MDV reactivation without significant induction of apoptosis and necrosis. In summary, fluorescent protein expression during latency reactivation is a robust tool to examine viral replication in live cells *ex vivo*, and temperature treatment is an efficient technique to induce reactivation without cytotoxicity effects on cell viability seen with chemical treatment.

Finally, an interesting case report by Suárez-Santana et al., documented the first report of disease caused by the Buteo buteo herpesvirus infection in the common buzzard (*Buteo buteo insularum*) from Gran Canaria (Canary Islands, Atlantic Ocean). Buteo buteo herpesvirus belongs to the Alphaherpesvirinae subfamily (18). The major lesions in the infected bird included necrotizing heterophilic and histiocytic bilateral conjunctivitis, stomatitis, pharyngitis, rhinitis, and sinusitis with secondary bacterial and fungal infections. They also observed eosinophilic intranuclear inclusion bodies in the oral mucosa and esophagus epithelium.

In summary, this Research Topic highlights the latest research in animal herpesvirus-host interactions, emerging virus infections, novel mobile genetic elements associated with herpesviruses, emerging therapeutics, and new *ex vivo* models that will facilitate further study of herpesvirus infection.

## Author contributions

SP: Writing—original draft. WA: Writing—review & editing.

## References

[1] McGeoch DJ Davison AJ. The descent of human herpesvirus 8. Semin Cancer Biol. (1999) 9:201–9. 10.1006/scbi.1999.0093 10343071

[2] McGeoch DJ Cook S. Molecular phylogeny of the alphaherpesvirinae subfamily and a proposed evolutionary timescale. J Mol Biol. (1994) 238:9–22. 10.1006/jmbi.1994.1264 8145260

[3] Adler B Sattler C Adler H. Herpesviruses and their host cells: a successful liaison. Trends Microbiol. (2017) 25:229–41. 10.1016/j.tim.2016.11.009 27956142

[4] Azab W Dayaram A Greenwood AD Osterrieder N. How host specific are herpesviruses? Lessons from herpesviruses infecting wild and endangered mammals. Annu Rev Virol. (2018) 5:53–68. 10.1146/annurev-virology-092917-043227 30052491

[5] Grinde B. Herpesviruses: latency and reactivation - viral strategies and host response. J Oral Microbiol. (2013) 5:1–9. 10.3402/jom.v5i0.22766 24167660 PMC3809354

[6] Pavulraj S Kamel M Stephanowitz H Liu F Plendl J Osterrieder N. Equine herpesvirus type 1 modulates cytokine and chemokine profiles of mononuclear cells for efficient dissemination to target organs. Viruses. (2020) 12:999. 10.3390/v12090999 32911663 PMC7551999

[7] Kamel M Pavulraj S Fauler B Mielke T Azab W. Equid herpesvirus-1 exploits the extracellular matrix of mononuclear cells to ensure transport to target cells. iScience. (2020) 23:101615. 10.1016/j.isci.2020.101615 33015592 PMC7521387

[8] Engels M Ackermann M. Pathogenesis of ruminant herpesvirus infections. Vet Microbiol. (1996) 53:3–15. 10.1016/S0378-1135(96)01230-8 9010994

[9] Maes R. Felid herpesvirus type 1 infection in cats: a natural host model for alphaherpesvirus pathogenesis. ISRN Vet Sci. (2012) 2012:495830. 10.5402/2012/495830 23762586 PMC3671728

[10] Cavignac Y Esclatine A. Herpesviruses and autophagy: catch me if you can! *Viruses* (2010) 2:314–33. 10.3390/v2010314 21994613 PMC3185561

[11] Liu X Cohen JI. The role of PI3K/Akt in human herpesvirus infection: from the bench to the bedside. Virology. (2015) 479–480:568–77. 10.1016/j.virol.2015.02.040 25798530 PMC4424147

[12] Oladunni FS Horohov DW Chambers TM. EHV-1: a constant threat to the horse industry. Front Microbiol. (2019) 10:2668. 10.3389/fmicb.2019.02668 31849857 PMC6901505

[13] Pusterla N Hussey GS Goehring LS. Equine herpesvirus-1 myeloencephalopathy. Vet Clin North Am Equine Pract. (2022) 38:339–62. 10.1016/j.cveq.2022.05.006 35811201

[14] Frost LS Leplae R Summers AO Toussaint A. Mobile genetic elements: the agents of open source evolution. Nat Rev Microbiol. (2005) 3:722–32. 10.1038/nrmicro1235 16138100

[15] Inoue Y Saga T Aikawa T Kumagai M Shimada A Kawaguchi Y. Complete fusion of a transposon and herpesvirus created the Teratorn mobile element in medaka fish. Nat Commun. (2017) 8:551. 10.1038/s41467-017-00527-2 28916771 PMC5601938

[16] Bertzbach LD Conradie AM You Y Kaufer BB. Latest insights into Marek's disease virus pathogenesis and tumorigenesis. Cancers. (2020) 12:647. 10.3390/cancers12030647 32164311 PMC7139298

[17] Denesvre C Rémy S Trapp-Fragnet L Smith LP Georgeault S Vautherot JF. Marek's disease virus undergoes complete morphogenesis after reactivation in a T-lymphoblastoid cell line transformed by recombinant fluorescent marker virus. J Gen Virol. (2016) 97:480–6. 10.1099/jgv.0.000354 26612074 PMC5394746

[18] Žlabravec Z Slavec B Vrezec A Kuhar U Zorman Rojs O Golob Z. Detection of herpesviruses in wild bird casualties in Slovenia. Front Vet Sci. (2022) 9:822212. 10.3389/fvets.2022.822212 35280151 PMC8916610

